# Prevalence of virulence and antimicrobial resistance genes in *Salmonella* spp. isolated from commercial chickens and human clinical isolates from South Africa and Brazil

**DOI:** 10.4102/ojvr.v83i1.1067

**Published:** 2016-05-26

**Authors:** Oliver T. Zishiri, Nelisiwe Mkhize, Samson Mukaratirwa

**Affiliations:** 1 Department of Genetics, University of KwaZulu-Natal, South Africa

## Abstract

Salmonellosis is a significant public health concern around the world. The injudicious use of antimicrobial agents in poultry production for treatment, growth promotion and prophylaxis has resulted in the emergence of drug-resistant strains of *Salmonella*. The current study was conducted to investigate the prevalence of virulence and antimicrobial resistance genes from *Salmonella* isolated from South African and Brazilian broiler chickens as well as human clinical isolates. Out of a total of 200 chicken samples that were collected from South Africa 102 (51%) tested positive for *Salmonella* using the *InvA* gene. Of the overall 146 *Salmonella*-positive samples that were screened for the *iroB* gene, most of them were confirmed to be *Salmonella enterica* with high prevalence rates. All the *Salmonella* isolates obtained were subjected to antimicrobial susceptibility testing with ten antibiotics. *Salmonella* isolates from South African chickens exhibited resistance to almost all antimicrobial agents used. All the samples were further subjected to the Polymerase Chain Reaction in order to screen some common antimicrobial and virulence genes of interest, namely *spiC, pipD, misL, orfL, pse-1, tet A, tet B, ant (3”)-la, sul 1* and *sul.* All the *Salmonella*-positive isolates exhibited resistance to at least one antimicrobial agent; however, antimicrobial resistance patterns demonstrated that multiple drug resistance was prevalent. The findings provide evidence that broiler chickens are colonised by pathogenic *Salmonella* harbouring antimicrobial resistance genes. Therefore, it is evident that there is a need for prudent use of antimicrobial agents in poultry production systems in order to mitigate the proliferation of multiple drug resistance across species.

## Introduction

The increasing human population around the world places a huge demand on food in order to ensure the survival of mankind. This exerts pressure on a number of food industries such as poultry production systems, where growth promotion agents are used in an effort to satisfy the increasing food demand. The presence of *Salmonella* in chicken meat and its related products has often caused them to be unsafe for human consumption (Centers for Disease Control [CDC] 2013). *Salmonella* is classified as one of the most common zoonotic foodborne pathogens that cause outbreaks and sporadic cases of gastroenteritis in humans throughout the world (Humphrey 2000). In the United States of America a total of 19 531 infections, 4563 hospitalisations and 68 deaths associated with foodborne diseases were reported in 2012 (CDC 2013). Epidemiological studies have reported numerous times that foods of animal origin, particularly poultry, are major vehicles associated with illnesses caused by *Salmonella* (Dallal *et al*. 2010). *Salmonella* can grow as surface-associated aggregates on food surfaces and equipment (Chia *et al*. 2009), commonly described as biofilms. The cells that develop as biofilms are potential sources of contamination on food products, which can result in infection in human hosts (Chia *et al*. 2009).

The reservoir of *Salmonella* is the gastro-intestinal tract (GIT) of a wide range of domestic and wild animals, and a variety of foodstuffs of both animal and plant origins are potential sources of infection (Thong *et al*. 2002; Thorns 2000). According to the Centers for Disease Control and Prevention (CDC) the genus *Salmonella* is divided into two species, *Salmonella enterica* and *Salmonella bongori* (CDC 2013). The species *S. enterica* is further subdivided into six subspecies that are designated by taxonomic names such as *S. enterica* subsp. *enterica, S. enterica* subsp. *salamae, S. enterica* subsp. *arizonae, S. enterica* subsp. *diarizonae, S. enterica* subsp. *houtenae* and *S. enterica* subsp. *indica* (CDC 2013). *Salmonella* is associated with approximately 2500 serovars. These serovars are separated based on differences in their lipopolysaccharide layer with regard to their somatic (O) and flagellar (H) antigens (Amagliani, Brandi & Schiavano 2012). With regard to the O antigen, *Salmonella* is divided into 50 serogroups and then further divided into more than 2500 serovars based on the H antigens present (Amagliani *et al*. 2012). The majority of the serovars of *Salmonella* belong to *S. enterica* and the most common serovar associated with zoonotic infection is *Salmonella enteriditis*, followed by *Salmonella typhimurium* (Amagliani *et al*. 2012). Serovars that are generally found in food products of animal origin include *S. enteriditis, S. typhimurium, Salmonella gallinarum, Salmonella weltevreden* and *Salmonella infantis* (Foley *et al*. 2011). Salmonellosis in both humans and animals results from various vehicles of *Salmonella* serovars such as *S*. *enteritidis, S*. *infantis, Salmonella kentucky*, and *Salmonella Heidelberg.* These vehicles that cause infection appear to be more prevalent in poultry than in any other food animal (Foley *et al*. 2011).

The ability of bacteria to infect the host relies on the genetic determinants called virulence genes, located in the *Salmonella* pathogenicity islands (SPI). According to Groisman and Ochman (1997), SPIs are portions of DNA that have been acquired from other microorganisms by horizontal gene transfer and they are absent in non-pathogenic strains. At least 60 virulence genes associated with SPIs (Groisman & Ochman 1997) have been mapped so far and they all serve different functions. Some facilitate colonisation for the pathogen to survive host defence mechanisms and some are responsible for multiplication inside the host. However, during contamination the host infection outcomes depend on various factors such as age, environment and genetics to influence the host status (Van Asten & Dijk 2005). A well-known virulence gene in *Salmonella* is the *Salmonella* invasion gene A (*invA*), which is responsible for host invasion (Galan, Ginocchio & Costeas 1992). This gene is vital because it is conserved in all *Salmonella* and is found in SPI-1. Hence it is used by researchers as a marker to detect *Salmonella* isolated from different sources.

Various studies from both developed and developing countries have focused on investigating the prevalence of genes encoding for virulence in *Salmonella*. These countries include the United States of America (Zou *et al*. 2011), Senegal and Gambia (Dione *et al*. 2011), Brazil (Borges *et al*. 2013; Dias De Olivieira *et al*. 2003) and Nigeria (Smith *et al*. 2015). In South Africa information on the prevalence of virulence genes in *Salmonella* of animal and human origin is limited. Therefore, these among other facts motivated us to embark on the current study in which we evaluated the prevalence of virulence genes in *Salmonella* such as *InvA, spiC, pipD, misL* and *orfL*. The Sip proteins, which include *SipA, SipB, SipC* and *SipD*, were some of the first virulence genes to be characterised in *Salmonella* based on their involvement in the invasion of cultured epithelial cells (Kaniga, Trollinger & Galan 1995). *SipC* is directly involved in the translocation process and is delivered to the cytosol of the host cell, where it may also have an effector function (Hersh *et al*. 1999). The *SpiC* gene was investigated in this study because it is involved in the interaction with the intercellular membrane and trafficking, thereby hindering correct cellular function (Uchiya & Nikai 2008). The *misL* is an SPI-3 encoded protein that is involved in other aspects of pathogenesis such as chronic infection and host specificity (Blanc-Potard *et al*. 1999). The *orfL* virulence gene is found in SPI-4 and is involved in adhesion, autotransportation and colonisation (Hughes *et al*. 2008). Another important virulence gene is the *pipD*, which is found in SPI-5 and is a type III secreted effector associated with the SPI-1 system (Hughes *et al*. 2008).

The use of antimicrobial agents in poultry production for treatment purposes, growth promotion and prophylaxis raises a major concern with regard to antimicrobial resistance and multidrug resistance, which are frequently observed among many *Salmonella* serovars (Duong *et al*. 2006). Increasing evidence demonstrates that antimicrobial usage in animals promotes the emergence of a wide range of resistant zoonotic pathogens such as *Salmonella*, which compromises the effectiveness of antibiotic treatments used in humans when an infection occurs (Gyles 2008). The variety of antibiotics that are administered in veterinary practice therapeutically has caused selective pressure, resulting in an increase in genetic sequences that confer resistance on microorganisms. Antimicrobial-resistant *Salmonella* has been recognised as a public health concern for decades in developed and developing countries and the evolving resistance in this pathogen limits the therapeutic options available to physicians for the treatment of human salmonellosis (Foley *et al*. 2011). Extensive use of antibiotics in chicken production systems for non-therapeutic purposes such as growth promotion results in the resistance of bacteria to these antimicrobial agents. Bacteria use both natural and acquired resistance mechanisms to protect themselves against agents that could harm them. Acquired resistance arises from mutations, gene transfer by conjugation or transformation, transposons, integrons and bacteriophages (Cogliani, Goossens & Greko 2011).

It is therefore necessary to determine bacterial resistance to antibiotics of all classes, the phenotypes they exhibit and the mutations responsible for resistance using molecular genetic analysis methods. The impact of antimicrobial resistance on human health is of great concern for the treatment of various infections that arise from food of animal origin (Glenn *et al*. 2011), where combinations of broad-spectrum antibiotics need to be administered in order to control infection. Therefore, understanding the mechanisms of antibiotic resistance, the location of genes on a chromosome or plasmid and their expression will assist in developing screening and control strategies that are urgently needed in order to reduce the spread and evolution of resistant bacteria. Against this background, this study aimed to investigate the prevalence of virulence and antimicrobial resistance genes in chicken samples from South Africa as well as imports from Brazil and human clinical isolates. Genetic characterisation of the antimicrobial resistance and virulence genes present in *Salmonella* is essential in understanding the pathogenicity and prevalence of resistance that exists in this zoonotic foodborne pathogen.

## Materials and methods

### Sample collection

Broiler chicken caecum samples were collected on the day of slaughter from poultry slaughterhouses within the Durban metropolitan area in KwaZulu-Natal province of South Africa between March and October 2014. Samples were collected in batches of 25 per month. In total 200 samples were randomly collected over the eight-month period. All the samples were aseptically collected in plastic screw-top tubes containing 45 mL of 0.1% w/v peptone-water and stored on ice until transported back to the University of Kwa-Zulu Natal (Westville Campus), where enrichment of the samples was done immediately on arrival. South Africa imports more than 50% of chicken products from Brazil because domestic production cannot meet the current demand. Whenever chicken products are imported in batches from other countries such as Brazil, quality assurance, routine disease surveillance, screening and testing are conducted before the products are conveyed to the food chain. It was therefore crucial to include some samples that originated from Brazil in our study. Accordingly, *Salmonella* isolates from Brazilian broiler chickens (24) and human clinical samples (20) from patients emanating from the coastal region of KwaZulu-Natal province in South Africa were provided by the National Health Laboratory Service of South Africa.

#### Enrichment

Enrichment was carried on South African (SABC) chicken samples only. Ten millilitres of rinse peptone-water from the samples were incubated at 37 °C for 24 h. After incubation 0.1 mL aliquots from the peptone-water samples were inoculated into tubes containing 10 mL of Rappaport Vassiliadis (RV) broth medium and incubated at 42 °C for 48 h (Ahmed & Shimamoto 2012).

#### Microbiological analysis

After enrichment, a loopful of the broth culture was streaked onto xylose-lysine-deoxycholate (XLD) agar plates and incubated at 37 °C for 24 h. Black colonies with typical phenotypic characteristics were regarded as suspect *Salmonella*. Suspected *Salmonella* colonies were picked and inoculated on TSB broth and incubated while shaking at 37 °C for 24 h. The resulting culture was used for DNA extraction and some was used for susceptibility tests. The remaining culture was used for 60% glycerol stocks that were then stored at -80 °C for future purposes.

#### DNA extraction

Genomic DNA of all the *Salmonella* isolates was extracted from the culture using the ZymoResearch Fungal and Bacterial Genomic DNA MiniPrep^TM^ kit following the manufacturer’s instructions. A positive *Salmonella* control was prepared by isolating genomic DNA from a reference strain of known *Salmonella* broth culture. After DNA extraction, a NanoDrop Spectrophotometer was used to check the concentration and quality of the isolated DNA and extracted DNA was then stored at -20 °C until use in Polymerase Chain Reaction (PCR).

### Confirmation of *Salmonella* using Polymerase Chain Reaction

PCR was performed on the DNA extracted from all the detected and obtained samples. The *invA* gene was used to confirm the presence of *Salmonella*. A 25 µL PCR reaction was used for amplification of the *invA* gene. The primers used for detection of the *invA* gene are presented in Table 1. The PCR reaction was carried out in a total volume of 25 µL containing 12.5 µL DreamTaq Green PCR Master Mix, 1 µL *invA* primer (forward), 1 µL *invA* primer (reverse), 4 µL of template DNA and 6.5 µL dH_2_O. Amplification was carried out in a thermo-cycler using 34 cycles consisting of denaturation for 30 s at 95 °C, annealing for 30 s at 58 °C, extension for 1 min at 72 °C and final extension for 5 min at 72 °C. PCR products were run on a 1.5% agarose gel using electrophoresis at 70 volts for 60 min to detect a 284 base pair product size of the *invA* target gene. Furthermore, the *iroB* gene that is unique to *S. enterica* species was used to confirm the identity of the species. The primers used are presented in Table 1 and everything was done following the same procedure used for *invA* gene amplification, except for the annealing temperature, which in this case was 55 °C for 40 s.

**TABLE 1:** Primers used to confirm *Salmonella* spp.

Target gene	Primer sequence (5’→ 3’)	Product size (bp)	Reference
*invA*^a^	F:TCATCGCACCGTCAAAGGAACC R:GTGAAATTATCGCCACGTTCGGGCAA	284	Li *et al*. (2012)
*iroB*^b^	F:TGC GTA TTC TGT TTG TCG GTCC R:TAC GTT CCC ACC ATT CTT CCC	606	Baumler *et al*. (1997)

Note: Please see the full reference list of the article, Zishiri, O.T., Mkhize, N. & Mukaratirwa, S., 2016, ‘Prevalence of virulence and antimicrobial resistance genes in *Salmonella* spp. isolated from commercial chickens and human clinical isolates from South Africa and Brazil’, *Onderstepoort Journal of Veterinary Research* 83(1), a1067. http://dx.doi.org/10.4102/ojvr.v83i1.1067, for more information.

a used to confirm all *Salmonella* spp.

b used to confirm *Salmonella enterica.*

### Detection of virulence genes

The primers used to detect virulence genes are depicted in Table 2. The PCR reactions was carried out in a total volume of 25 µL and under the following conditions: *spiC* gene (initial denaturation at 94 °C for 12 min, 1 min of denaturation at 94 °C, 30 s of annealing at 54 °C and 5 min of extension at 72 °C for a total of 34 cycles; 5 s were added to the extension time each cycle); *misL* and *orfL* genes (3 min at 94 °C, 35 cycles of 1 min at 94 °C, 1 min at 58 °C and 1 min at 72 °C and finally 5 min at 72 °C) and for *pipD* gene (94 °C for 5 min, 34 cycles of 25 s of denaturation at 94 °C, 30 s of annealing at 56 °C and 50 s of extension at 72 °C and a final cycle at 5 min at 72 °C). Gel electrophoresis of amplified products was then carried out in 1.5% agarose in a 1X TBE buffer containing GelRed. After the gels were run, PCR products were visualised using the ChemiDoc^TM^ imaging system.

**TABLE 2:** Primers used to detect virulence genes in *Salmonella* spp.

Target gene	Primer sequence (5’ → 3’)	Size (bp)	Annealing temperature	Mechanism of resistance	Broad action
*spiC*	F:CCTGGATAATGACTATTGAT R:AGTTTATGGTGATTGCGTAT	309	54	Type III secretion system	Survival in macrophages
*misL*	F:GTCGGCGAATGCCGCGAATA R:GCGCTGTTAACGCTAATAGT	400	60	Involved in intramacrophage survival	Survival in macrophages
*orfL*	F:GGAGTATCGATAAAGATGTT R:GCGCGTAACGTCAGAATCAA	550	60	Adhesin/autotransporter	Colonisation
*pipD*	F:CGGCGATTCATGACTTTGAT R:CGTTATCATTCGGATCGTAA	350	56	Type III secretion effector associated with SPI-1 system	Enteritis

*Source*: Hughes, L.A., Shopland, S., Wigley, P., Bradon, H., Leatherbarrow, A.H., Williams, N.J. *et al*., 2008, ‘Characterisation of *Salmonella enterica* serotype Typhimurium isolates from wild birds in northern England from 2005–2006’, *Journal of Infectious Disease in Developing Countries* 4, 4. http://dx.doi.org/10.1186/1746-6148-4-4

### Antimicrobial susceptibility testing

Antimicrobial resistance of the 146 *Salmonella* isolates were tested against ten antimicrobial agents using the Kirby-Bauer disc diffusion method on Mueller Hinton Agar following the guidelines of the Clinical and Laboratory standards Institute (CLSI) (CLSI 2008). The antimicrobials selected were those commonly used in the poultry industry, namely gentamicin (10 µg), amoxicillin (10 µg), erythromycin (10 µg), chloramphenicol (30 µg), tetracycline (10 µg), trimethoprim (1.25 µg), ampicillin (10 µg), streptomycin (25 µg), trimethoprim-sulfamthoxazole (25 µg) and kanamycin (30 µg). The Oxoid antibiotic discs were impregnated with the concentrations of each antibiotic. First, Mueller Hinton Agar was inoculated with 0.1 mL of nutrient broth samples, which had been inoculated with a loopful of glycerol stocks of positive samples and then incubated at 37 °C for 24 h. With the use of a glass hockey stick, the culture was spread onto the agar for even distribution of the organisms that demonstrated the presence of *Salmonella* after PCR, thereafter; discs impregnated with antibiotics were evenly placed on plates and then incubated at 37 °C for 24 h. The inhibition zones were measured and scored as sensitive (S), intermediate susceptibility (I) and resistant (R) according to the CLSI recommendations. *Escherichia coli* ATCC 25922 was used as a reference strain for antibiotic disc control (Bacci *et al*. 2012).

### Detection of antimicrobial resistance genes

Genomic DNA of *Salmonella* extracted was used to detect antimicrobial resistance genes. The primer sets used to detect antimicrobial resistance genes are presented in Table 3. The PCR reactions were carried out in total volumes of 25 µL each using the following conditions: *pse-1* gene (initial denaturation at 94 °C for 12 min, 1 min of denaturation at 94 °C, 30 s of annealing at 57 °C and 5 min of extension at 72 °C for a total of 34 cycles; 5 s was added to the extension time each cycle); *ant (3”)-la* gene (3 min at 94 °C, 35 cycles of 1 min at 94 °C, 1 min at 58 °C and 1 min at 72 °C and finally 5 min at 72 °C); *tet A* and *tet B* gene (94 °C for 5 min, 34 cycles of 25 s of denaturation at 94 °C, 30 s of annealing at 55 °C and 50 s of extension at 72 °C and a final cycle at 5 min at 72 °C). *Sul1* and *sul2* detection was carried in the same way as for tetracycline genes, but with annealing temperatures of 65 °C and 52 °C respectively. Gel electrophoresis of the amplified products was then carried out in 1.5% agarose in a 1X TBE buffer containing GelRed. After the gels were run, the PCR products were visualised using the ChemiDocTM imaging system.

**TABLE 3:** Primers used to screen antimicrobial resistance genes in *Salmonella* spp.

Antimicrobial agent	Target gene	Primer sequence (5’–3’)	Size (bp)	References	Mechanism of resistance
Ampicillin	*pse-1*	F:CGCTTCCCGTTAACAAGTAC R:CTGGTTCATTTCAGATAGCG	419	Bacci *et al*. (2012)	-
Gentamicin	*ant (3”)-la*	F:GTGGATGGCGGCCTGAAGCC R:ATTGCCCAGTCGGCAGCG	526	Bacci *et al*. (2012)	Aminoglycoside adenyltransferase
Tetracycline	*tet A*	F:GCTACATCCTGCTTGCCTTC R:CATAGATCGCCGTGAAGAGG	210	Bacci *et al*. (2012)	Efflux
	*tet B*	F:TTGGTTAGGGGCAAGTTTTG R:GTAATGGGCCAATAACACCG	659	Bacci *et al*. (2012)	Efflux
Sulfamethoxazole	*Sul 1*	F:GCG CGG CGT GGG CTA CCT R:GATTTCCGCGACACCGAGACAA	350	Poppe *et al*. (2006)	Dihydropteroate synthase inhibitor
	*Sul 2*	F:CGG CAT CGT CAA CAT AACC R:GTG TGC GGA TGA AGT CAG	720	Poppe *et al*. (2006)	Dihydropteroate synthase inhibitor

## Ethical approval

The animal studies were approved by the appropriate ethics committee of the University of KwaZulu-Natal (Reference: 012/15/Animal); therefore, they have been performed in accordance with the ethical standards laid down in the 1964 Declaration of Helsinki and its later amendments.

## Results

### Culture identification

**FIGURE 1:**
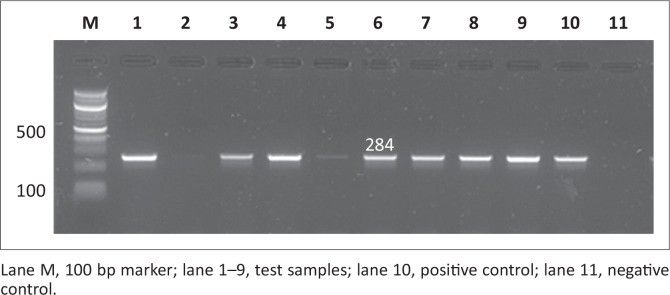
Detection of the 284 bp *invA* gene amplicon from eight representative *Salmonella* isolates by agarose gel electrophoresis.

**FIGURE 2:**
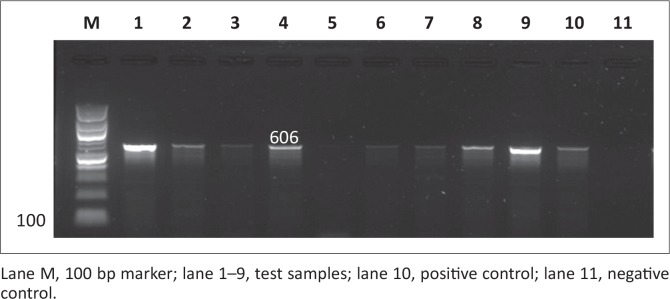
Detection of the 606 bp *iroB* gene amplicon from nine representative *Salmonella* isolates by agarose gel electrophoresis.

Out of the 200 samples collected from SABC slaughterhouses, only 102 (51%) were confirmed positive for *Salmonella*. The 102 *Salmonella* isolates together with the 24 *Salmonella* isolates from Brazilian chickens (BBC) and 20 human clinical isolates (SAHC) obtained from National Health Laboratory Service (NHLS) made the total of samples used in this study 146. Out of the 146 samples screened for the *iroB* gene, most were confirmed to be *Salmonella enterica* with the following prevalence rates: 17 (85%) of human clinical samples, 70 (68.6%) of South African chicken samples and 17 (70.8%) of Brazilian chicken samples. Figures 1 and 2 illustrate the amplification PCR products for *invA* and *iroB* gene respectively.

### Detection of virulence genes in *Salmonella* spp.

**FIGURE 3:**
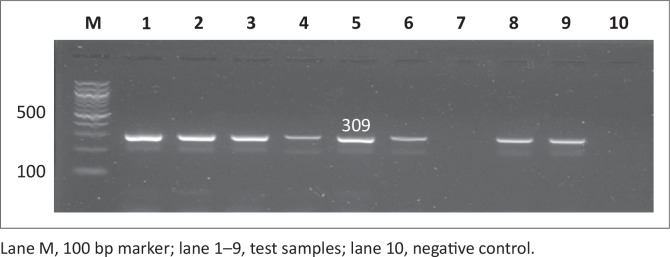
Detection of the 309 bp *spiC* gene amplicon from eight representative *Salmonella* isolates by agarose gel electrophoresis.

**FIGURE 4:**
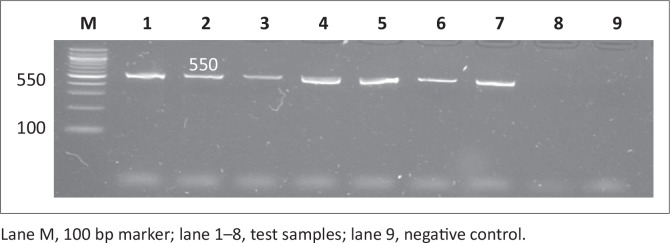
Detection of the 550 bp *misL* gene amplicon from seven representative *Salmonella* isolates by agarose gel electrophoresis.

**FIGURE 5:**
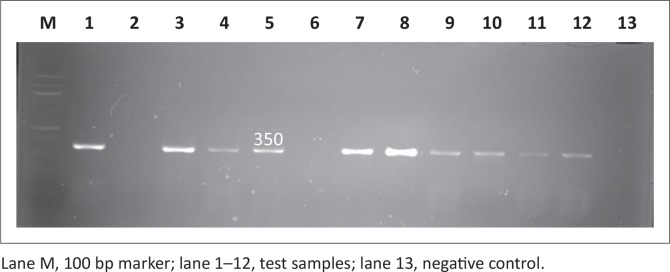
Detection of the 350 bp *orfL* gene amplicon from nine representative *Salmonella* isolates by agarose gel electrophoresis.

**FIGURE 6:**
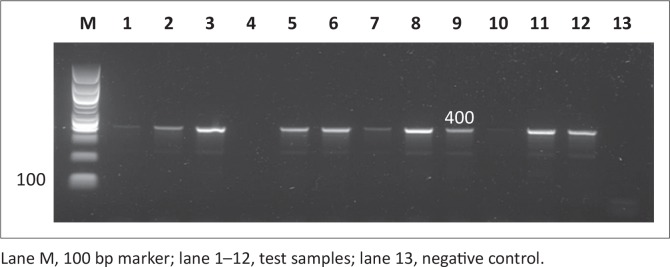
Detection of the 400 bp *pipD* gene amplicon from nine representative *Salmonella* isolates by agarose gel electrophoresis.

PCR was used to screen for all four virulence genes and all the genes screened were depicted in Figures 3, 4, 5 and 6. All the amplicon sizes were consistent with the sizes that were expected. Table 4 shows that *spiC* (47%), *pipD* (35%), *misL* (2%) and *orfL* (20.6%) genes were harboured in South African broiler chicken isolates. It was also demonstrated that Brazilian broiler *Salmonella* isolates harboured all the genes, with the following prevalence rates: *spiC* (83%), *pipD* (87.5%), *misL* (29%) and *orfL* (25%). The same table presents the results for South African human clinical isolates that were harbouring 85% of *spiC* gene, followed by *pipD* (80%), then *misL* (75%), and lastly 20% of *orfL*.

**TABLE 4:** Prevalence of virulence genes in *Salmonella* isolates from three different origins.

Origin	Virulence genes												

	Number of isolates (*n*)	*invA*	%	*sipC*	%	*pipD*	%	*misL*	%	*orfL*	%	*iroB*	%
SABC	102	102	100	48	47	36	35	2	2	21	20.6	70	68.6
BBC	24	24	100	20	83	21	87.5	7	29	6	25	17	70.8
SAHC	20	20	100	17	85	16	80	15	75	10	20	17	85
Total	146	146	100	85	58	73	50	24	16	37	25	104	71.2

SABC, South African broiler chicken isolates; BBC, Brazilian broiler chicken isolates; SAHC, South African human clinical isolates.

### Antimicrobial susceptibility testing

A total of 146 *Salmonella* isolates from different origins were tested for resistance to ten antimicrobial agents using the disc diffusion method. The incidences of resistance for all the isolates tested are presented in Table 5. *Salmonella* isolated from SABC exhibited resistance to all ten antimicrobials and the highest rates of resistance observed were: tetracycline (93%), trimethoprim-sulfamthoxazole (84%), trimethoprim (78.4%), kanamycin (74%), gentamicin (48%), ampicillin (47%), amoxicillin and chloramphenicol (31%), erythromycin (18%) and streptomycin (12%). Isolates from Brazilian broiler chickens also demonstrated resistance to all antimicrobials tested and 100% of the isolates exhibited complete resistance to ampicillin, amoxicillin and tetracycline (83%), trimethoprim (66.7%), erythromycin (62.5%), trimethoprim-sulfamthoxazole (50%), kanamycin (16.7%), gentamicin and streptomycin (12.5%) and chloramphenicol (4.2%). *Salmonella* isolates from South African human clinical isolates exhibited resistance to eight antimicrobial agents. The highest resistance rates were observed for erythromycin and amoxicillin, both at 30%. Multidrug resistance was also observed across all isolates tested and Table 6 summarises the resistance patterns of *Salmonella* isolates in the current study. The resistance patterns provide evidence for multidrug resistance and from Table 6 it can be seen that the *Salmonella* isolates used have the potential to confer resistance to more than two antimicrobial agents.

**TABLE 5:** Antimicrobial susceptibility tests on *Salmonella* isolates of different origins.

Antibiotics	Number of isolates																	

	SABC†						BBC‡						SAHC§					
		
	R	%	I	%	S	%	R	%	I	%	S	%	R	%	I	%	S	%
AMP	48	47.0	41	40.0	13	13.0	24	100.0	0	0.0	0	0.0	3	15	8	40	9	45
AML	32	31.0	45	44.0	25	25.0	20	83.0	3	12.5	1	4.2	6	30	5	25	9	45
C	32	31.0	30	29.0	40	39.0	1	4.2	2	8.3	21	88.0	0	0	1	5	19	95
CN	49	48.0	26	26.0	27	26.5	3	12.5	3	12.5	18	75.0	0	0	0	0	20	100
E	18	18.0	13	13.0	71	69.6	15	62.5	6	25.0	3	12.5	6	30	10	50	4	20
K	75	74.0	9	9.0	18	18.0	4	16.7	0	0.0	20	83.0	1	5	0	0	19	95
S	12	12.0	8	8.0	82	80.0	3	12.5	15	62.5	6	25.0	4	20	3	15	13	65
SXT	86	84.3	0	0.0	16	15.7	12	50.0	4	16.7	8	33.0	3	15	0	0	17	85
TE	95	93.0	7	7.0	0	0.0	20	83.0	0	0.0	4	16.7	2	10	1	50	17	85
W	80	78.4	4	3.9	19	18.6	16	66.7	3	12.5	5	20.8	4	20	2	10	14	70

AMP, ampicillin; AML, amoxicillin; C, chloramphenicol; CN, gentamicin; E, erythromycin; K, kanamycin; S, streptomycin; STX, trimethoprim-sulfamthoxazole; TE, tetracycline; W, trimethoprim; SABC, South African broiler chicken isolates; BBC, Brazilian broiler chicken isolates; SAHC, South African human clinical isolates; R, Resistant; I, Intermediate susceptibility; S, Susceptible.

† *n* = 102;

‡ *n* = 24;

s§ *n* = 20.

**TABLE 6:** Antibiotic resistance patterns of *Salmonella* isolates illustrating multiple drug resistance.

Antibiotic resistance patterns	Number of isolates					

	SABC	%	BBC	%	SAHC	%
AMP, TE	32	31.4	20	83.0	1	5
AML, TE	31	30.4	16	66.7	2	10
TE, W	74	72.5	14	58.0	1	5
S, TE	12	11.8	3	12.5	0	0
K, TE	71	69.6	3	12.5	0	0
SXT, TE	69	67.6	10	41.7	0	0
E, TE	14	13.7	12	50.0	2	10
C, TE	19	18.6	1	4.2	0	0
AMP, AML, TE	30	25.0	16	80.0	1	5
AMP, C, TE	10	9.8	1	4.2	0	0
E, W	18	17.6	12	50.0	4	20
E, SXT, W	15	14.7	9	37.5	1	5
S, SXT, W	11	10.8	3	12.5	0	0
TE, W	74	72.4	14	58.0	1	5
AML, AMP, TE, W	23	22.5	12	50.0	0	0
AML, AMP, E, TE	12	11.8	10	41.7	0	0

AMP, ampicillin; AML, amoxicillin; C, chloramphenicol; CN, gentamicin; E, erythromycin; K, kanamycin; S, streptomycin; STX, trimethoprim-sulfamthoxazole; TE, tetracycline; W, trimethoprim; SABC, South African broiler chicken isolates; BBC, Brazilian broiler chicken isolates; SAHC, South African human clinical isolates.

### Molecular detection of antimicrobial resistance genes in *Salmonella*

**FIGURE 7:**
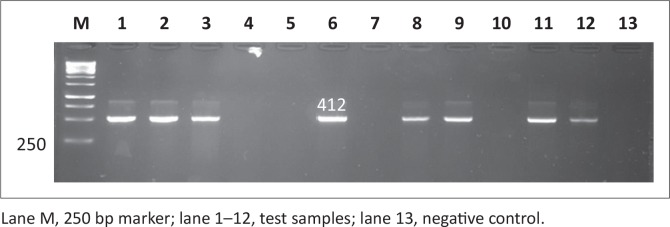
Detection of the 412 bp *pse-1* gene amplicon from eight representative *Salmonella isolates* by agarose gel electrophoresis.

**FIGURE 8:**
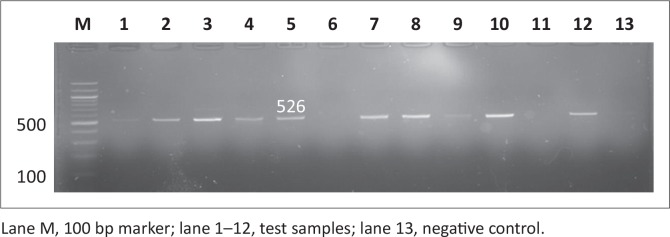
Detection of the 526 bp *ant (3”)-la* gene amplicon from eight representative *Salmonella* isolates by agarose gel electrophoresis.

**FIGURE 9:**
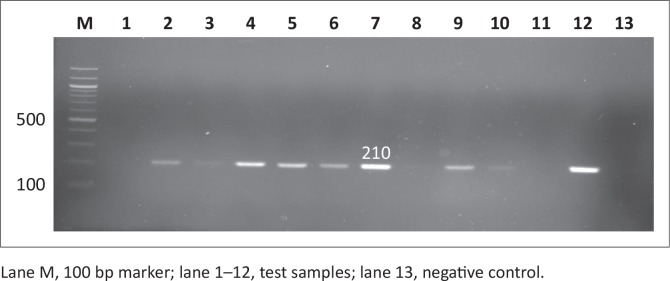
Detection of the 210 bp *tet A* gene amplicon from seven representative *Salmonella* isolates by agarose gel electrophoresis.

**FIGURE 10:**
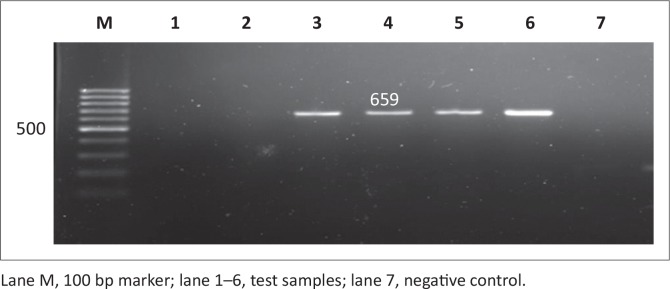
Detection of the 659 bp *tet B* gene amplicon from four representative *Salmonella* isolates by agarose gel electrophoresis.

**FIGURE 11:**
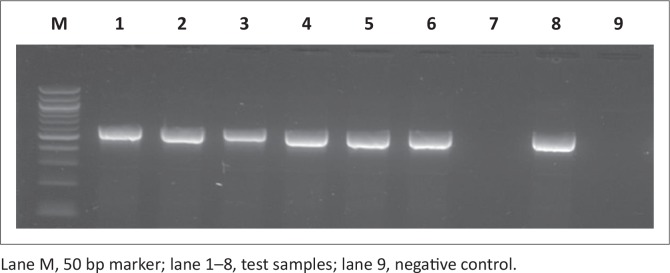
Detection of the 350 bp sul 1 gene amplicon from seven representative Salmonella isolates by agarose gel electrophoresis.

**FIGURE 12:**
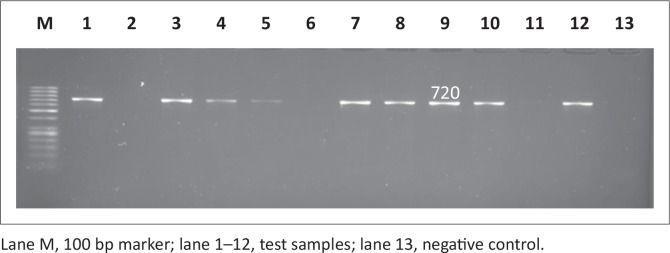
Detection of the 720 bp *sul 2* gene amplicon from eight representative *Salmonella* isolates by agarose gel electrophoresis.

Antimicrobial resistance genes were detected in all 146 *Salmonella* isolates, regardless of antimicrobial susceptibility, using the disc diffusion method since this method is presumptive and needs to be supplemented with other tests. Observed PCR results indicated that detected *Salmonella* contained antimicrobial resistance genes known to confer resistance. Six antimicrobial resistance genes were screened in total: *pse-1, ant (3”)-la, tet A, tet B, sul 1* and *sul 2*; the phenotypes of the genes are illustrated in Figures 7, 8, 9, 10, 11 and 12 respectively. The prevalence rates of the genes detected are presented in Table 7. In the SABC isolates the most prevalent antimicrobial resistance gene that was detected was *pse-1* gene (56%), known to confer resistance to ampicillin. This gene was followed by *tet A* (44%), *ant (3”)-la* (32%) and *tet B* (28%), known to confer resistance to tetracycline, gentamicin and tetracycline respectively as depicted in Table 7. In the Brazilian broiler chicken isolates *tet A* and *sul 1* genes (83%) were the most prevalent genes, followed by *sul 2* (79%), *ant (3”)-la* (75%), *pse-1* (63%) and *tet B* (33%). Finally, in the South African human isolates the gene that showed the highest prevalence was *ant (3”)-la* (80%), followed by *tet A* (70%). The *tet B, sul 1* and *sul 2* genes all exhibited 60% prevalence and lastly, the *pse-1* gene reported a (50%) prevalence. These results are partially consistent with the antimicrobial susceptibility testing because most of the genes were detected in isolates that exhibited resistance.

**TABLE 7:** Prevalence of antimicrobial resistance genes screened from 146 *Salmonella* isolates.

Antibiotics	Resistance genes	Number of isolates					

		SABC†	%	BBC‡	%	SAHC§	%
Ampicillin	*pse-1*	57	56.0	15	63	10	50
Gentamicin	*ant (3”)-la*	33	32.0	18	75	16	80
Sulfamethoxazole	*sul 1*	44	43.0	20	83	12	60
	*sul 2*	43	42.0	19	79	12	60
	*sul 1, sul 2*	18	17.6	18	75	10	50
Tetracycline	*tet A*	45	44.0	20	83	14	70
	*tet B*	29	28.0	8	33	12	60
	*tet A, tet B*	15	14.7	6	25	7	35

† *n* = 102;

‡ *n* = 24;

§ *n* = 20.

## Discussion

The injudicious use of antimicrobials leads to resistance in various bacteria such as *Salmonella*, and therefore antibiotic resistance in foodborne bacterial enteric pathogens is an almost inevitable consequence of the irrational use of antimicrobial drugs in animal production systems (Newell *et al*. 2010). Findings from the current study demonstrated the presence of *Salmonella* in broiler chicken isolates at farm level. Out of the 200 samples tested for *Salmonella* only 102 (51%) samples tested positive using PCR, amplifying the *invA* gene. These results were almost in agreement with results presented in previous studies (Antunes *et al*. 2003; Capita, Alonso-Calleja & Prieto 2007; Chuanchuen & Padungtod 2009; Hao Van *et al*. 2007), which reported *Salmonella* prevalence as 57%, 49%, 60% and 53.3% respectively. Cortez *et al*. (2006) conducted a study on the identification of *Salmonella* isolates from chicken abattoirs and reported that out of 288 samples collected 52 (18%) samples were tested positive. These results are relatively low and contrary to results obtained from the current study. Moreover, similar previous studies (Dogru *et al*. 2010; Kaushik *et al*. 2014; Van Nierop *et al*. 2005; Zewdu & Cornelius 2009) also reported very low *Salmonella* prevalence – 8%, 23.7%, 19.2% and 8% respectively – compared to the current study. Information from the literature provides proof that broiler chickens are potential carriers of *Salmonella*. However, various factors contributing to the high prevalence of *Salmonella* isolates were detected in the present study. Feed, housing and the hygiene status of the farms where the chickens were reared may be some of the factors. The presence of *Salmonella* species at farm level is a serious concern because it shows that the potential exists for the pathogens to disseminate from the farms to communities. This is of great concern because the majority of South Africans depend on food sold in informal retail outlets where hygienic conditions are questionable and promote the accumulation and proliferation of pathogens, posing a danger to consumers. The presence of *Salmonella* in chicken meat is quite serious because South Africa imports a significant amount of poultry products from other countries such as Brazil, China and the USA. Consumers are therefore at risk of contracting salmonellosis from either local or imported poultry products.

It is worth noting that the greatest attribute *Salmonella* uses to survive in a host cell is pathogenicity. Various studies have probed the significance of pathogenicity in a pathogen–host interaction and most concluded that pathogenicity is indeed significant for the pathogen to survive and proliferate. Pathogenicity helps *Salmonella* to invade and destroy epithelial cells in the host intestines (Uchiya & Nikai 2008) and then to colonise other cell lines. All this happens as a result of the presence of the genetic determinants responsible for virulence in *Salmonella* spp.

In the current study the prevalence of four virulence genes was established. The virulence genes were selected on the basis of their functions and danger to chickens and humans. All the genes detected belonged to different *Salmonella* pathogenicity islands (SP1). It was not part of the scope of the study to detect these islands, but the information was sourced from the literature. *Salmonella* pathogenicity islands SP1-2, SP1-5, SP1-3 and SP1-4 encode for *spiC, pipD, misL* and *orfL* genes respectively.

The function of the *spiC* gene is to interact with intercellular membrane trafficking in such a manner as to alter it, hindering the correct cellular functioning (Uchiya & Nikai 2008). The results obtained from South African broiler chickens present s*piC* gene as the most prevalent virulent gene that was detected among the four genes that were screened. The prevalence rates reported were 47%, 35%, 2%, and 20.6%, all of them corresponding to the following genes respectively: *spiC, pipD, misL* and *orfL*. The detection of these genes in isolates obtained at a farm level on the carcasses of healthy chickens emphasises the fact that healthy chickens with no sign of illness can be carriers of pathogenic *Salmonella* (Skyberg, Logue & Nolan 2006). This demonstrates the risk of chicken meat to consumers and implies the possible dissemination of virulence genes.

The presence of the same virulence genes in human clinical samples demonstrates the dissemination and distribution of virulence genes, although the origin of the *Salmonella* that infected the patients was not known because no background information had been obtained from patients about possible sources of infection with *Salmonella*. *Salmonella* isolates detected from Brazilian imported chicken meat also demonstrated the presence of all the virulence genes screened during the study, with *spiC* and *pipD* genes as the most prevalent, having a prevalence rate of 83% and 87% respectively. The genes with the lowest prevalence rates were *orfL* (25%) and *misL* (29%); both have been reported to be responsible for the survival of *Salmonella* in host macrophages.

Virulence genes have also been detected in previous studies from Brazil (Borges *et al*. 2013; Castilla *et al*. 2006; Dias de Olivieira *et al*. 2003), West Africa (Dione *et al*. 2011), Colombia (Sánchez-Jiménez *et al*. 2010) and England (Hughes *et al*. 2008). Therefore, the detection of virulence genes from chicken meat imported from other countries and then sold in South Africa indicates the possibility of a transfer of *Salmonella* pathogenic strains from other countries to South Africa. Although virulence genes are common in local *Salmonella* strains, receiving foreign strains can worsen the situation by increasing the prevalence of genes encoding for pathogenicity and increasing genetic diversity of *Salmonella* strains in South Africa.

Antimicrobial resistance is a global public health problem. The increasing antimicrobial resistance in *Salmonella* is a forward irreversible reaction, but it can be reduced if certain precautions are followed worldwide. Tetracycline is a commonly used antimicrobial agent in human and animal medicine because it is cheap and easily accessible. Tetracycline resistance has been reported worldwide, and it comprises three types of resistance mechanism, namely tetracycline efflux, tetracycline modification and ribosomal protection (Roberts 2005). During the current study *Salmonella* isolates from Brazilian chicken isolates, South African chicken isolates and South African human isolates exhibited resistance to tetracycline, with prevalence rates of 83%, 93% and 10% respectively. Both genes known to confer resistance to tetracycline, namely *tet A* and *tet B*, were detected in some isolates that exhibited resistance. The prevalence of *tet A* was higher than *tet B* in all the groups of isolates screened. This was quite similar to the findings in previous studies that reported the same pattern (Chuanchuen & Padungtod 2009; Miko *et al*. 2005). Not all of the confirmed *Salmonella* isolates were harbouring *tet A* and *tet B* genes, leading to the prevalence of the genes being low compared to the prevalence of *Salmonella* resistance to tetracycline.

These results imply that there might be other antimicrobial genes conferring resistance that were not detected in the study, as there are other determinants that confer resistance to tetracycline, namely *tet C, tet D, tet R, tet M* and several others. However, in the human isolates the prevalence of resistance was very low, but the two genes were detected even in the isolates that were susceptible to tetracycline.

Ampicillins and amoxicillin are among the drugs of choice for treating salmonellosis (De Toro *et al*. 2011). In the current study, *Salmonella* isolates from Brazilian chickens demonstrated high resistance to these two drugs compared to other isolates. A gene conferring resistance to β-lactamase, namely *pse-1,* was detected in most of the *Salmonella* isolates found to be resistant to ampicillin and amoxicillin. Llanes, Kirchgesner and Plesiat (1999) reported that resistance to β-lactam is due to the production of the *pse-1* enzyme. According to Glenn *et al*. (2011), *Salmonella* spp. isolated from food-producing animals have been reported to carry the *pse-1* gene and it is one of the most prevalent β-lactamases.

The current study demonstrated high prevalence of *Salmonella* isolates with the *pse-1* gene compared to similar studies (Bacci *et al*. 2012; Chuanchuen & Padungtod 2009) that reported very low prevalence rates of 0% and 5% respectively. South African studies have detected the presence of *pse-1* gene in aquatic systems and in livestock production (Igbinosa 2015; Igbinosa & Okoh 2012). Although studies related to the mapping *pse-1* gene have been conducted, there is still a paucity of information on the prevalence of this gene in South Africa. More information on such genes can contribute to the solution of developing new drugs. Since high prevalence rates of *pse-1* gene were observed, this implies that the presence of β-lactamase in foodborne pathogens is increasing. Batchelor, Hopkins and Threlfall (2005) speculated that the increasing presence of β-lactamase in pathogenic bacteria limits the therapeutic use of antimicrobial agents. The *ant (3”)-la* gene is one of the aminoglycoside resistance determinants. It has been detected in a number of bacterial pathogens, but information about this gene’s prevalence in *Salmonella* is limited, especially in Africa, where little research has been done. In the current study it was detected in some of the gentamicin-resistant *Salmonella* isolates. Moreover, genes conferring resistance to sulfamethoxazole (*sul 1* and *sul 2*) were also detected in most *Salmonella* isolates that exhibited resistance to trimethoprim-sulfamethoxazole. The results showed that some isolates were even harbouring both genes.

Multidrug resistance is an increasing problem that has been reported in both animal and human medicine. *Salmonella* isolates used in the present study illustrated a high rate of multidrug resistance. The results presented in Table 6 show patterns illustrating multidrug resistance, which can be confirmed if an isolate is resistant to more than two antibiotics. There may be several possible explanations for such outcomes but the main one could be a lack of compliance with legislation governing the amount and type of antimicrobial agents used in the South African poultry industry and also in human medicine. Multidrug resistance has a negative impact on therapy in both animal and human medicine. Various studies have proven that infections caused by multidrug-resistant *Salmonella* strains are more dangerous than the infections caused by susceptible strains, since they extensively delay therapy, placing patients’ lives at risk (Martin *et al*. 2004; Varma *et al*. 2005). The presence of multidrug-resistant *Salmonella* strains in chickens and also in humans has serious implications for public health systems and for the economy.

Overall, the results obtained from the study demonstrate that the detected *Salmonella* strains harboured both virulence and antimicrobial resistance genes. The potential exists for the random dissemination of these genes, with negative implications for the health and welfare of chickens and humans and could result in increased antimicrobial resistance. Since some developed and developing countries have prohibited the use of some antimicrobial agents as feed additives in animal husbandry, especially poultry, South Africa should take note of what has been happening in other countries with regard to the regulation of antimicrobial use and attempt to prevent the escalating antibiotic resistance problem.

## Conclusion

In conclusion, all of the 12 genes examined in this study were successfully amplified in the *Salmonella* isolated from different origins. These findings indicate that the selective pressure caused by the variety of antibiotics administered therapeutically in veterinary practice and poultry production systems for growth promotion and prophylaxis has resulted in an increase in genes conferring resistance to *Salmonella*. It is difficult to make comparisons between the *Salmonella* surveillance surveys conducted in different countries as the prevalence of *Salmonella* varies regionally and isolation rates depend upon the country, sample plan and methodology used. The data from this study indicate the dissemination of antimicrobial resistance genes in *Salmonella* isolated from broiler chickens at the abattoir level. The emergence and dissemination of antimicrobial-resistant *Salmonella* in food animals has major public health implications, especially for large-scale suppliers who export their products both regionally and internationally. Therefore, foodborne salmonellosis should constantly be monitored.

Future work in the area of this study should include organism specificity by serotyping positive *Salmonella* samples in order to determine the serovars of *Salmonella* which are most prevalent in broiler chickens. Furthermore, phylogenetic analyses should also provide interesting insights into determining how closely related the positive *Salmonella* isolates are to each other.
